# Supplementary material for: Repellency and toxicity of long-lasting insecticide-treated bed nets (LLINs) to bed bugs
Source: PLoS One. 2025 Jan 3;20(1):e0313595. doi: 10.1371/journal.pone.0313595 (PMC11698412; doi:10.1371/journal.pone.0313595)
Supplement: Attachment — Submitted filename: PONE-D-24-35489_ResponsetoReviewers.docx [file pone.0313595.s002.docx]

**Response to reviewers**

Please note that “line ##” refers to the revised “clean” version (no track-changes) of the manuscript.

**Reviewer 1**

The Title can be adapted as " Repellency and toxicity of various types of long-lasting insecticide-treated bed nets to bed bugs".

Response #1: We thank the reviewer for the suggestion. We have changed the title to “Repellency and toxicity of long-lasting insecticide-treated bed nets (LLINs) to bed bugs”.

Abstract and Introduction started with malaria which does reflect well the research topic, these can be explained later in the text but not at the beginning.

Response #2: Given the nature of this project we respectfully disagree. LLINs are fundamental components of malaria control programs. The findings we present are critically significant in the context of malaria control, and therefore this context should be presented to the reader from the beginning of the manuscript. We introduce vector control as a critical intervention in malaria control and immediately shift to LLINs which are pivotal tactics in vector control. We do likewise in the Introduction.

Line 59: Suggested to write the full name of DEET at least for the first time in the text.

Response #3: We replaced “DEET” with “N,N-diethyl-meta-toluamide” in line 59 of the manuscript.

Lines 130-134: Possible to rephrase the sentences from line 130 to 134 to make it more clear.

Response #4: We revised the suggested sentences to better represent the preparation process.

Lines 195 and others : 12h to 96h or 12h till 96h?

Response #5: We revised this section to clarify that mortality was recorded every 12 h until 96 h. See lines 194-195 & 380.

Figures 2, 3, & 5: For figure 2, 3 and 5, figure description is too long. Part of it can be included in text of the Result section.

Response #6: We thank the reviewer for this comment. Given the complexity of the figures, we feel it is important to include as much information in the figure legends as possible to allow readers to fully comprehend all aspects of the figure without needing to repeatedly refer to the text in the M&M and Results sections. Nevertheless, we removed from figure legends the definition of Activation and reference to % Activate and % Choice. We also removed the definition of mortality and the ambient conditions from Fig 5.

Line 454: the proposed hypotheses could be common for all types of LLINs, described in the following paragraphs is good.

Response #7: We thank the reviewer for this comment.

Discussion: In discussion, 80% mortality in Fuller Mill strain exposed to all types of LLIN after 4 days is not clear from fig 5B as different survival ratios are shown after 96 hours.

Response #8: The PermaNet 3.0R line (purple “dash-dot-dot-dash”) indicates a proportion surviving of 0.2 at 96 h for the Fuller Mill strain, indicative of 80% mortality at 96 h.

**Reviewer 2**

Abstract: Line 15-16: Success of vector control programs hinges on community acceptance of products like long-lasting insecticide-treated nets (LLINs). Suggestion: As you have not answered “Community acceptance of the product”, You should not raise the question.

Response #9: The wording of this sentence accurately reflects evidence within the literature which demonstrates association between product efficacy and community acceptance. See Malede *et al.* 2019 (ref #13) and review by Hayes & Schal 2024 (ref #7).

Line 209: Word “Lack of” should not be used in the title, because it is your finding

Response #10: We respectfully disagree. We have framed the subheadings throughout the Results section to summarize the respective Results section in a single phrase to focus the reader’s attention and highlight the key takeaways.

Line 277: Word Marginal should not be used in the title, because it is your finding

Response #11: Please see Response #10.

Line 332: Similarly, word No should not be used.

Response #12: Please see Response #10.
